# A 180 Myr-old female-specific genome region in sturgeon reveals the oldest known vertebrate sex determining system with undifferentiated sex chromosomes

**DOI:** 10.1098/rstb.2020.0089

**Published:** 2021-08-30

**Authors:** Heiner Kuhl, Yann Guiguen, Christin Höhne, Eva Kreuz, Kang Du, Christophe Klopp, Céline Lopez-Roques, Elena Santidrian Yebra-Pimentel, Mitica Ciorpac, Jörn Gessner, Daniela Holostenco, Wibke Kleiner, Klaus Kohlmann, Dunja K. Lamatsch, Dmitry Prokopov, Anastasia Bestin, Emmanuel Bonpunt, Bastien Debeuf, Pierrick Haffray, Romain Morvezen, Pierre Patrice, Radu Suciu, Ron Dirks, Sven Wuertz, Werner Kloas, Manfred Schartl, Matthias Stöck

**Affiliations:** ^1^ Department of Ecophysiology and Aquaculture, Leibniz-Institute of Freshwater Ecology and Inland Fisheries (IGB), Müggelseedamm 301 and 310, 12587 Berlin, Germany; ^2^ INRAE, LPGP, 35000 Rennes, France; ^3^ Developmental Biochemistry, Biocenter, University of Würzburg, 97074 Würzburg, Germany; ^4^ The Xiphophorus Genetic Stock Center, Department of Chemistry and Biochemistry, Texas State University, San Marcos, TX 78666, USA; ^5^ SIGENAE, Plate-forme Bio-informatique Genotoul, Mathématiques et Informatique Appliquées de Toulouse, INRAe, 31326 Castanet-Tolosan, France; ^6^ INRA, US 1426, GeT-PlaGe, Genotoul, 31326 Castanet-Tolosan, France; ^7^ Future Genomics Technologies B.V., Sylviusweg 74, 2333 BD, Leiden, The Netherlands; ^8^ Danube Delta National Institute for Research and Development, Tulcea 820112, Romania; ^9^ Genetic Improvement Laboratory, Research Station for Cattle Breeding Dancu - Iasi (SCDCB Dancu), Academy of Agricultural and Forestry Sciences ‘Gheorghe Ionescu-Sisesti’, Iasi-Ungheni Street, No. 9, Holboca, Iași county 707252, Romania; ^10^ Research Department for Limnology, University of Innsbruck, A-5310 Mondsee, Austria; ^11^ Institute of Molecular and Cellular Biology, Siberian Branch of the Russian Academy of Sciences, 630090 Novosibirsk, Russia; ^12^ SYSAAF, Station INRAE-LPGP, Campus de Beaulieu, 35042 Rennes cedex, France; ^13^ L'Esturgeonnière, Allée de Mios, 33470 Le Teich, France; ^14^ SCEA Sturgeon, 29 rue du Carillon, 17240 Saint Fort sur Gironde, France

**Keywords:** acipenseridae, sturgeon, sex chromosomes, female-specific, polyploidy, evolution

## Abstract

Several hypotheses explain the prevalence of undifferentiated sex chromosomes in poikilothermic vertebrates. Turnovers change the master sex determination gene, the sex chromosome or the sex determination system (e.g. XY to WZ). Jumping master genes stay main triggers but translocate to other chromosomes. Occasional recombination (e.g. in sex-reversed females) prevents sex chromosome degeneration. Recent research has uncovered conserved heteromorphic or even homomorphic sex chromosomes in several clades of non-avian and non-mammalian vertebrates. Sex determination in sturgeons (Acipenseridae) has been a long-standing basic biological question, linked to economical demands by the caviar-producing aquaculture. Here, we report the discovery of a sex-specific sequence from sterlet (*Acipenser ruthenus*). Using chromosome-scale assemblies and pool-sequencing, we first identified an approximately 16 kb female-specific region. We developed a PCR-genotyping test, yielding female-specific products in six species, spanning the entire phylogeny with the most divergent extant lineages (*A. sturio, A. oxyrinchus* versus *A. ruthenus, Huso huso*), stemming from an ancient tetraploidization. Similar results were obtained in two octoploid species (*A. gueldenstaedtii, A. baerii*). Conservation of a female-specific sequence for a long period, representing 180 Myr of sturgeon evolution, and across at least one polyploidization event, raises many interesting biological questions. We discuss a conserved undifferentiated sex chromosome system with a ZZ/ZW-mode of sex determination and potential alternatives.

This article is part of the theme issue ‘Challenging the paradigm in sex chromosome evolution: empirical and theoretical insights with a focus on vertebrates (Part I)’.

## 1. Introduction

Sexual reproduction is an old feature of life. In vertebrates, sexual development is determined by environmental triggers (environmental sex determination, ESD) or genotypic sex determination (GSD) (e.g. [1]) or a combination thereof. In an existing GSD-system, the paradigmatic evolutionary model of sex chromosome evolution suggests the rise of a new sex chromosome from an autosome by mutation or translocation of a gene, which becomes a trigger of the sex-determining regulatory networks. Heterozygotes encode one sex and homozygotes the other [2–4]. According to this classical view, mutations and/or linkage to genes with sexually antagonistic effects may be favoured near this gene, i.e. mainly in unpaired parts of Y and W chromosomes, profiting from linkage disequilibrium. To preserve epistasis, recombination between such mutations and the sex-determining locus would then be suppressed in the heterogametic sex [5,6]. Such non-recombining sex chromosomes are expected to degenerate through the progressive accumulation of deleterious mutations [4,7], except for small pseudo-autosomal regions. Recently, as an alternative or additional cause of degeneration, instability and divergence of *cis*-regulatory sequences in non-recombining genome regions, which become selectively haploidized to mask deleterious mutations on coding sequences, have been suggested [8].

In contrast to the conservation of differentiated sex chromosomes in most mammals and birds, in the majority of poikilothermic vertebrates differentiated sex chromosomes are rarely observed [9–11] (but see [12]). Several hypotheses have been offered to explain the prevalence of undifferentiated sex chromosomes. The high-turnover model proposes that the emergence of new master sex-determining genes on new chromosomes would prevent sex chromosome degeneration [13–16]. The ‘jumping sex locus’ hypothesis [17] implies that the sex determination gene is maintained but changes its position to different chromosomes, preventing evolutionary decay of the original sex chromosome [18]. The ‘fountain of youth’ hypothesis [19] suggests that sex chromosomes may escape from degeneration by occasional recombination. Because sex-specific recombination depends on phenotypic rather than genotypic sex, homomorphic X and Y chromosomes might recombine in sex-reversed females. These rare events should generate bursts of new Y haplotypes, which will be quickly sorted out by natural or sexual selection [19]. Seemingly young sex chromosomes may thus carry old-established sex-determining genes, challenging the opinion that sex chromosomes unavoidably decay [20,21].

Beyond mammals and birds, conserved sex chromosomes have recently been discovered in several amniote (specifically reptile) clades [22–25], all of which feature differentiated sex chromosomes. Evolutionarily very old, conserved and homomorphic ZZ/ZW sex chromosomes are known in some ratite birds (Ratidae), dating back more than 130 Myr [26,27]. Similarly, skinks (Scincidae) share homologous, mostly poorly differentiated XX/XY sex chromosomes across a wide phylogenetic spectrum for at least 85 million years [28]. Recent findings in the teleost family Esocidae report undifferentiated sex chromosomes of similar evolutionary age (65–90 Myr) in teleosts [29]. The Salmonidae family, which experienced a whole-genome duplication *ca* 90 Ma [30], presumably harbours a conserved sex determination gene, perhaps as old as 50 Myr, on different chromosomes in different species [17,18], which encodes a homomorphic XX/XY sex chromosome system. Here we report the discovery of a sex-specific region in sturgeon (Acipenseridae), which is preserved in many extant species with a common ancestor about 180 Ma.

Sturgeons and paddlefish comprise 27 living species [31], which branched off 330 Ma [32] from the root of the more than 31 000 living teleosts. The evolutionary history of vertebrates is characterized by several rounds of whole-genome duplications (WGD). The 1R and 2R WGD happened early on, before the jawed vertebrates appeared, but the sturgeon branch diverged before the teleost 3R WGD [33,34]. Later on, sturgeon experienced several clade-specific polyploidizations, resulting in chromosome numbers from approximately 120 up to 380 [35]. Based on a chromosome-scale genome assembly of *Acipenser ruthenus*, Du *et al.* [32] have recently shown a whole genome sequencing (WGS), early in the sturgeon radiation. Although subsequent rediploidization of the genome caused the loss of entire chromosomes or large fragments (i.e. segmental deduplication), structural and functional tetraploidy was maintained over 180 Myr, and a decrease of redundancy in the tetraploid genome follows mostly random processes. Sturgeons possess undifferentiated microscopically indistinguishable sex chromosomes [32,36]. Although the sex ratio of offspring from experimental gynogenesis led to the assumption that all sturgeons possess female-heterogametic (ZZ/ZW) sex chromosome systems [37], a sex-specific marker, confirming female heterogamety and allowing genetic studies, has not been found [32].

## 2. Material and methods

### (a) Animal sampling and establishment of genetic families

#### (i) Acipenser ruthenus

For pool-sequencing (pool-seq), 61 (31 female, 30 male) adult sterlets from the broodstock at the Leibniz-IGB (Berlin, Germany), representing the Danube River population [32], were sexed by gonadal biopsies, evidence from reproduction and a few autopsies. In addition, 52 sterlets (table 1) were sampled from three aquaculture populations. These came from two other *A. ruthenus* brood stocks, originating from the Danube (Wöllershof, Bavaria, Germany, 20–25 cm, *ca* 1 year old; and Beucha, Saxony, Germany, 10–12 cm, six months old), as well as *A. ruthenus* interpopulation hybrids from the Volga and Danube Rivers (12–20 cm, 8–10 months). They were euthanized and gonadal sex determined histologically, and finclips used for genotyping. In spring 2020, a genetic family was derived from the IGB-broodstock; at the age of four months, histological sections of gonads from 14 offspring were prepared, and finclips from them and their parents also used for genetics.

**Table 1.** Numbers of individuals tested with the sex-linked PCR-marker *AllWSex2* (*p*-values for association with sex were calculated using a Pearson's *χ*^2^-test with Yates' continuity correction; gel pictures in electronic supplementary material, figures S3–S10). To avoid circular evidence, neither the 61 (31 females, 30 males) *A. ruthenus* used for pool-sequencing, nor the offspring of the three genetic families of *A. ruthenus, A. oxyrhinchus* and *H. huso* (electronic supplementary materials, figures S8–S10) are counted here, but only their parents.

species	ploidy	positive females	positive males	*p*-value of association with sex	type of phenotypic sexing
*A. sturio*	4n	18/18	0/18	1.456 × 10^−8^	ultrasound, biopsy if contradiction to genotype, few autopsies
*A. oxyrinchus*	4n	12/12	0/15	1.536 × 10^−6^	adult breeders with known reproduction, histology
*H. huso*	4n	12/12	0/16	9.311 × 10^−7^	adult breeders with known reproduction, biopsy, histology
*A. ruthenus*	4n	25/25	0/27	4.11 × 10^−12^	adult breeders with known reproduction, biopsy, histology
*A. baerii*	8n	28/28	0/28	5.352 × 10^−13^	adult breeders with known production of gametes
*A. gueldenstaedtii*	8n	15/15	0/12	1.536 × 10^−6^	adult breeders with known reproduction, ultrasound, few autopsies

#### (ii) Huso huso

Using minimal invasive biopsy samples from the gonads of 28 subadult and adult beluga sturgeon from two fish farms (Horia and Peceneaga, Romania, fish between 10 and 13 years old) were inspected by the unaided eye and/or microscopically after standard haematoxylin/eosin staining (described below). Finclips were sampled for genetic analyses (table 1). To generate genetic families, on 6 April 2018, a beluga sturgeon male (PIT-tag 000004691858; 200 cm, approx. 100 kg) was captured in the Danube δ region at river-km 126 (distance from the Black Sea) in Romania. On 13 April 2018, a mature *H. huso* female was caught (PIT 642099000568452; total length (TL) 260 cm, approx. 150 kg, near km 100), along with another male on 14 April 2018 (PIT 968000004692054; TL 143 cm, approx. 22 kg, km 126). All three were transferred to an aquaculture company (Horia, Romania). After stimulation of all the three wild-caught animals and one additional captive male with gonadotropin-releasing hormone (GnRH), ovulation started on April 16. Eggs were fertilized with sperm from each male separately. Fin clips of the four adult fish were taken, and the offspring of the individual matings were reared separately until the age of one year. All offspring fish were individually tagged with PIT-tags and then reared in common tanks. For this study, fish of one genetic family were sampled at the age of 15–24 months for gonadal histology and finclips. Wild-caught adults and, after 2 years, hundreds of their tagged offspring were released into the Danube to minimize the impact on this highly threatened species.

#### (iii) Acipenser oxyrinchus

Tissue samples of 27 adult Baltic sturgeon (table 1) from the broodstock in Mecklenburg-Pomerania (Born, Germany), kept for re-introduction into Oder River and Baltic Sea, were taken from tagged males and females and sexed using gonadal biopsies and evidence from reproduction. In 2017, genetic families were derived from breeding pairs during the yearly reproduction. Finclips from the parental animals were kept in ethanol. A subset of juveniles was raised in separate tanks at the Leibniz-IGB (Berlin, Germany) for 3 years.

#### (iv) Acipenser sturio

Tissue samples of 36 adult European sturgeon (table 1) from a broodstock of immature fish at the IGB, raised for conservation aquaculture to re-introduce the species into the Elbe River, were initially sexed using ultrasonic evidence. Gonadal biopsies were taken if genotyping did not match the ultrasonic evidence. Few specimens came from autopsy of deceased fish.

#### (v) Acipenser baerii

Finclips of 56 adult Siberian sturgeon (table 1) used as breeders with known phenotypic sex were sampled in an aquaculture breeding company (l'Esturgeonnière, France) and kept in 100% ethanol until DNA extraction.

#### (vi) Acipenser gueldenstaedtii

Finclips of 27 adult Russian sturgeons (table 1) were sampled in an aquaculture breeding company (Ecloserie de Guyenne, SCEA Sturgeon, France) and kept in 100% ethanol until DNA extraction. All fish were sexed based on evidence from ultrasound, reproduction and autopsy.

### (b) DNA processing for genotyping

DNA from finclips or other tissue samples was extracted using the DNeasy Tissue Kit (Qiagen) or the BioSprint robotic workstation with the 96 DNA Plant Kit (Qiagen, Germany), according to the manufacturer's protocols.

### (c) Histological sexing of juvenile and subadult fish as well as offspring of genetic families

Offspring from genetic families of *H. huso, A. ruthenus* and *A. oxyrinchus* were sacrificed by an overdose of buffered tricaine methanesulfonate (MS222; 0.3 g l^−1^, PHARMAQ); individual length and wet weight were recorded, finclips (stored in 100% ethanol) and gonadal samples were taken immediately. The right gonad strand was fixed in phosphate-buffered formaldehyde solution (ROTI^®^Histofix, 4%, Carl Roth) for at least 24 h at room temperature, then rinsed three times for 24 h with 70% ethanol, and stored at room temperature. Gonad strands were dehydrated in an increasing series of ethanol (75%, 90%, 95%, 100%), rinsed in xylol (Carl Roth) and transferred into Paraplast^®^ (Leica), using the Shandon Excelsior ES Tissue Processor (Thermo Fisher Scientific). Gonads were embedded in a spiral-like arrangement, to ideally cut the whole organ into 5 µm sections across its entire length (microtomes: Leica 2065 or Microm HM 340E, Thermo Fisher Scientific). Sections were mounted on glass slides and stained using standard haematoxylin/eosin (0.1%, Carl Roth). Histological evaluation was made at various magnifications using light microscope Nikon Eclipse Ni-U with Nikon DS-Fi3 camera, and the corresponding software Nikon DS-L4 to archive images.

### (d) Pool-sequencing

The genomic DNA of 31 female and 30 male *A. ruthenus* specimens was extracted from 90% ethanol-preserved fin clips using a classical phenol/chloroform protocol, quantified using Qubit fluorimetry and analysed using the Fragment Analyzer (Advanced Analytical Technologies, Inc., Iowa, USA). DNA short read sequencing was performed at the GeT-PlaGe core facility, INRAe Toulouse (https://get.genotoul.fr/en/). Two DNA pool-seq libraries were prepared according to manufacturer's protocols using the Illumina TruSeq Nano DNA HT Library Prep Kit (Illumina, California, USA). Briefly, from 200 ng of each sample, DNA was fragmented (550 bp) by sonication on a M220 Focused-ultrasonicator (COVARIS). Size selection was performed using SPB beads (kit beads) and 3′-ends of the blunt fragments were mono-adenylated. Then, adaptors and indexes were ligated and the construction amplified with Illumina-specific primers (eight cycles). Library-quality was assessed using Fragment Analyzer and libraries were quantified by qPCR using the Kapa Library Quantification Kit (Roche). Sequencing was performed on a NovaSeq S4 lane (Illumina, California, USA), using a paired-end read length of 2 × 150 bp with the Illumina NovaSeq Reagent Kits.

### (e) Analysis of sex-specific variants based on a male reference genome

Pool-seq data were mapped to the male *A. ruthenus* reference genome (*Acipenser_ruthenus*.ARUT1.2.dna.toplevel.fa, which is nearly identical to the current reference genome at NCBI GCF_010645085.1; electronic supplementary material, table S1) by Minimap2 (with parameters –t 12 –x sr –a; [38]) and converted to sorted bam-files by SAMtools [39]. Variants (SNPs, MNPs, INDELs) were called from both bam files using the Platypus variant caller [40] with integrated duplicate fragment reads removal (–filterDuplicates = 1) and reassembly of reads (–assembleAll = 1). The resulting vcf-file was screened for sex-specific variants using awk-scripting and different variant read coverage cutoffs (i.e. maximum reads in males (0, 1 … ), minimum reads in females (10, 13 … ) or *vice versa*). Variants were clustered according to their distance in the genome (maximal distance of variants in a cluster: 4000 bp), counted using BEDTools (merge, annotate; [41]) and sorted by variant count. Regions with highest variant densities and neighbouring regions were inspected for genes related to sex determination.

### (f) Reconstruction of male- and female-specific genomic regions from unmapped pool-seq data

Unmapped reads potentially representing regions not present in the reference genome were extracted from bam-files and assembled using the IDBA assembler [42]. The resulting male-/female-specific contigs were added to the reference genome and pool-seq data were mapped again (as described above) to this extended reference genome. Finally, sequencing coverage by female and male pools was compared in bedgraph files derived from the bam-files (bedtools genomecov –bga –split –ibam … ; bedtools unionbedgraph). These were screened for putative haploid coverage in one sex and no or low, spurious coverage in the other sex to identify female-/male-specific regions.

Another high-quality assembly of a female *A. ruthenus* has recently been published at NCBI by the Vertebrate Genomes Project (GCA_902713425.1). We repeated our analyses from above using this reference with an emphasis on detection of coverage differences. Using bedgraph-files, we compared female and male pool-sequencing coverage at the bp-level along the genome. We used different filtering approaches to call regions that had no or low coverage in one sex and approximately haploid coverage in the other sex. The most successful filtering applied awk-scripting to filter regions in male and female genomes where 0 coverage in male pool and a coverage of larger than 0.3 in the female pool were observed (here 1.0 was the normalized diploid coverage). These regions were combined in larger blocks if they were as close as 30 bp to each other (bedtools merge). Finally, we filtered for blocks that were larger than 160 bp and derived at a clear signal present only in female data and female genome.

### (g) Primer design and PCR-conditions

Using the male versus female *A. ruthenus* genome sequence as well as preliminary Illumina-data of a female *A. oxyrinchus* genome, we designed a number of primers that were primarily tested in gradient-PCRs on phenotypically sexed *A. ruthenus*. While several primers showed sex-specific amplification in this species, one (*AllWSex2*) could be optimized to work across multiple species (primer sequences and PCR-conditions: electronic supplementary material, texts S1–S3).

### (h) Phylogenomic reconstruction

To build a phylogenetic tree for sturgeons, we downloaded transcriptome assemblies of *A. baerii*, *A. oxyrinchus*, *Acipenser schrencki*, *Acipenser sinensis* and *Acipenser transmontanus* from the Public Sturgeon Transcripts Database (http://publicsturgeon.sigenae.org/home.html) and of *A. gueldenstaedtii* from NCBI (https://www.ncbi.nlm.nih.gov/Traces/wgs/?page=1&view=tsa&search=acipenser). The transcriptome of *Huso huso* was de novo assembled using Trinity [43] from mixed organs. Annotated genomes of *A. ruthenus* and *Latimeria chalumnae* were also downloaded from NCBI (electronic supplementary material, table S2). To identify orthologues between two species, we used the ‘Reciprocal best hit (RBH)’ method, and *A. ruthenus* as the hub to retrieve orthologous genes across all species. Protein sequences of the transcriptome contigs were predicted using GeneWise [44] by aligning each contig to its orthologous protein in *A. ruthenus*. Contigs with less than 60% of the protein aligned were discarded. In total, we collected 1017 genes with orthology across all species. For each orthologous gene, the protein sequences were aligned across all species using MAFFT [45], and trimmed them, using trimAl [46]. All 1017 alignments were then combined in a concatenated alignment, consisting of 383506 aligned amino acid sites. Finally, this alignment was transferred to RAxML [47] to construct a maximum-likelihood phylogeny.

## 3. Results

### (a) Analyses of sterlet (*Acipenser ruthenus*) female and male pool-sequencing

Pool-sequencing resulted in 553 117 774 reads (83.5 Gbp) and 486 762 084 reads (73.5 Gb) for the female and the male *A. ruthenus* pools, respectively. Pool-sequencing Illumina reads are available in the Sequence Read Archive (SRA: SRX9341540; SRX9341541), under BioProject reference PRJNA670610. After mapping to the reference genomes, the peak of the unimodal coverage distribution was 42× for the female pool and 37× for the male pool. There was no significant difference between both pools in the percentages of mapped reads (96.9% reads mapped).

We initially used two bioinformatic strategies to uncover female-specific pool-seq data in *A. ruthenus*. We first analysed sex-specific variants based on a male reference genome. The number of female- or male-specific variants was low, but their ratio already hinted at a ZW sex determination system (details: electronic supplementary material, text S4). In the top candidate region, which harboured 27 female-specific variants at HiC_scaffold_4:45 149 338–45 165 057 (electronic supplementary material, figure S1, table S1 and text S5), we found a female-specific 15 bp-indel, which we used to design PCR-primers. These amplified a product concordant with phenotypic sex of some *A. ruthenus*, but not all (data not shown). We also analysed male- or female-specific pool-seq reads, not matching the male reference genome, and independently assembled these unmapped reads from the male/female pools. This resulted in 1067 additional female-specific contigs spanning 658 732 bp. However, BlastX-searches using these contigs did not detect any sequences related to known sex determination genes.

### (b) Identification of a female-specific locus in *A. ruthenus* by coverage analysis using pool-seq data and a female reference genome

Due to a possible high repeat content of a W-locus, short-read assemblies might not be able to resolve it in large contigs but in rather fragmented small contigs, hindering further analysis. Recently, a long-read female haplotype reference of *A. ruthenus* has become available (GCA_902713425.1), which allowed remapping our pool-seq data and focusing on regions with different coverage between males and females. With stringent filtering parameters (no coverage in males; a minimum of 30% diploid coverage in females and minimum size of regions of 160 bp), we found 11 neighbouring matches (summing up to 2705 bp) in the female genome, while we did only find a single spurious match (185 bp) in the male genome. *Vice versa*, filtering for zero coverage in females and 30% of diploid coverage in males also resulted in no hits (female genome) or two spurious hits (175 and 181 bp; male genome).

**Figure 1.**
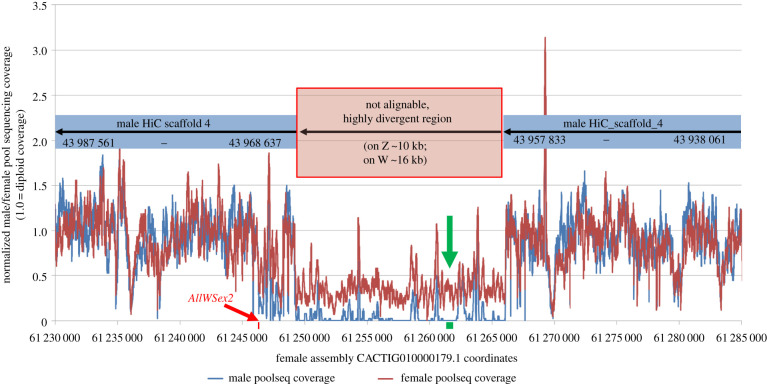
Detection of a female-specific locus in *Acipenser ruthenus*, when mapping female/male pool-sequencing reads to a female (ZW) reference genome assembly. Genome-wide signal detection was highly specific, when screening for larger regions (here ≥160 bp) with zero and non-zero coverage between sexes. The red block and arrow show the position of the *AllWSex2*-PCR-marker (CACTIG010000179.1: 61 246 236–61 246 344), which distinguishes sex in six sturgeon species. The green block and arrow mark the location of the only contig matching the region (CACTIG010000179.1: 61 261 166–61 261 703), when using the female-specific pool-seq read assembly approach.

The filtered regions in the female genome fell into the region CACTIG010000179.1:61 229 779–61 285 849 (including 20 kb up- and down-stream). This region corresponds to the region HiC_scaffold_4:43 938 061–43 987 561 in the male assembly as shown by the alignments of the 20 Kbp up- and down-stream sequences. Figure 1 illustrates the female and the male pool-seq coverage of the identified locus. The female-specific locus is located 1.162 Mb upstream of the identified cluster of 27 female-specific variants, mentioned above. Furthermore, a single contig (559 bp) of the 1067 female-specific contigs assembled from the female pool-seq data did match the identified locus. Thus, three independent analyses of the pool-seq data, specifically sequence variants, assembled female-specific reads and differential sequence coverage of the female genome assembly, support HiC_scaffold_4 as a sex chromosomal sequence, albeit with different specificity and resolution.

### (c) Establishment of a W-linked PCR across the sturgeon species tree

**Figure 2.**
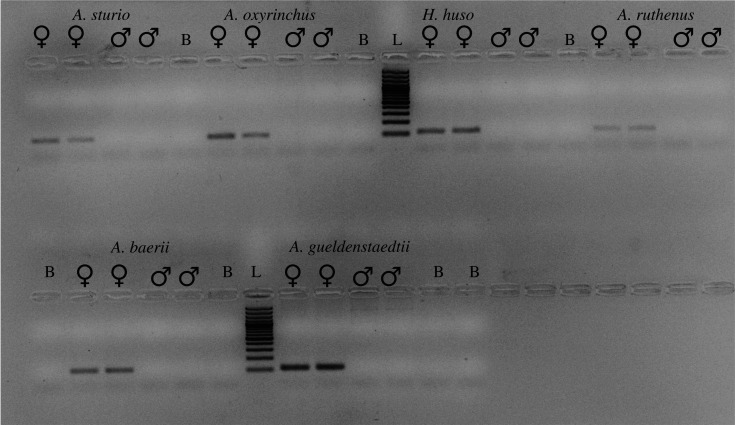
PCR-products using the *AllWSex2* marker in six species across the sturgeon phylogeny under identical PCR-conditions (electronic supplementary material, texts S1–S3) for two females and two males of each species. PCR-products migrated *ca* 45 min at 70 V on a 2% agarose gel in 1× TAE-buffer. Symbols and abbreviations: ♀: female; ♂: male; B: Blank, i.e. no DNA template in PCR; L: ladder, 100 bp-size-marker (Quantitas, Biozym).

**Figure 3.**
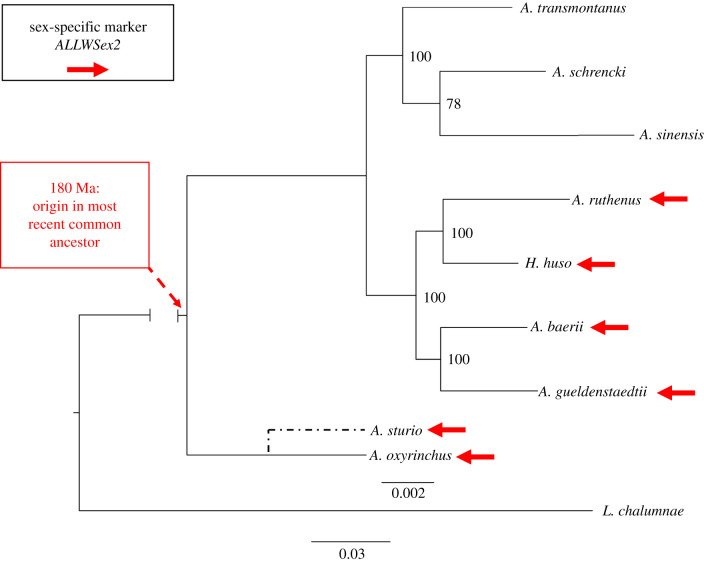
Phylogenomic tree of sturgeon species built by using RAxML with maximum-likelihood (for details, see §2). Numbers at nodes refer to bootstrap support values. The bar above the branch to *L. chalumnae* indicates branch length of sturgeon lineages, while the bar below the branch to *L. chalumnae* indicates branch length of the outgroup lineage coelacanth (*Latimeria chalumnae*). The approximate position of *A. sturio*, for which reliable transcriptomic data were unavailable, was added according to www.timetree.org. The root of Acipenseridae was dated at approximately 180 Ma, according to [32]. Red arrows mark those species for which we have obtained female-specific PCR-products, using primers *AllWSex2*_F/_R.

Our PCR-marker *AllWSex2* revealed female-specific products for all unambiguously phenotypically sexed females under identical PCR-conditions in six sturgeon species (table 1), spanning the entire phylogenetic tree and the most divergent extant lineages (*A. sturio, A. oxyrinchus* versus *A. ruthenus, H. huso*), all of which are tetraploid (approx. 120 chromosomes). The marker also showed sex-linkage in two octoploid species (*A. gueldenstaedtii* and *A. baerii*; table 1, figures 2 and 3).

### (d) Heredity of the sex-linked marker in three sturgeon species

In three genetic families, consisting of mother, father and their histologically sexed offspring, of *A. oxyrinchus*, *A. ruthenus* and *H. huso*, representing 180 Myr of sturgeon evolution, the marker *AllWSex2* was exclusively present in females, demonstrating mother to daughter transmission (gel pictures and histology in electronic supplementary material, figures S8–S16).

## 4. Discussion

We have discovered a female-specific (W) region, consistent with a potential ZZ/ZW system in sturgeon. This finding is in accordance with previous assumptions or indirect evidence that sturgeons exhibit a female-heterogametic sex determination system [36,48,49]. This genomic region is conserved in at least six sturgeon species, spanning the entire phylogenetic tree, represented by the most divergent species *A. sturio, A. oxyrinchus*, *A. ruthenus* and *H. huso*, all of which are formally ancient tetraploid (approx. 120 chromosomes) [32]. In the *A. ruthenus* genome, the sequence resides on chromosome 4 [32]. Of note, this genomic region is also sex-linked in two octoploid species (*A. gueldenstaedtii* with 258 ± 4 chromosomes, and *A. baerii* with 249 ± 5 chromosomes), demonstrating maintenance of this region and apparently a female-heterogametic sex determination system through at least one round of polyploidization. In the future, conservation and evolution of the region in the remaining extant sturgeons should be examined to evaluate the degree to which this ancient female-specific genomic region is also conserved in other species. In particular, identification of the master sex-determining gene(s) or other regulatory elements is required. At the current state of research, we also cannot rule out a situation like in Salmonidae, where a ‘jumping sex locus’, perhaps as old as 90 Myr [30], resides on different chromosomes in different species, all of which share a male heterogametic (XX/XY) system [17,18].

Our data reveal the oldest known vertebrate sex-determining system with undifferentiated sex chromosomes. Conservation of a female-specific sequence for 180 Myr of sturgeon evolution, and across at least one additional polyploidization event, raises many interesting biological and evolutionary questions. We hypothesize that this region might be part of the sex locus. Given the slow protein evolution of the sturgeon genome, evolving distinctly more slowly than in teleosts, including basal species such as arowana and arapaima, but also more slowly than gar, coelacanth or elephant shark [32], we speculate that the relatively conserved karyotypes of the ancient tetraploid species examined here (*A. sturio, A. oxyrinchus*, *A. ruthenus, H. huso*) may have even maintained the same undifferentiated sex chromosome for 180 Myr. It will be fascinating to examine the mechanisms that stabilized the sex determination system and whether or not these involve undifferentiated homologous sex chromosomes. Results from previous RADseq [32] and pool-sequencing of the same *A. ruthenus* (sampled from aquaculture populations, and with few if any family-effects expected), revealing 99.98% of the chromosome as largely undifferentiated, let us assume that the sturgeon sex chromosome contains a large pseudo-autosomal region (PAR; [50,51]; electronic supplementary material, figure S1). This and the large chromosome number (approx. 120–260 for the six species) make it challenging to apply SNPs or microsatellites to examine whether *AllWSex2* resides on the same or different linkage groups and therefore is beyond the framework of this study, although we have generated genetic families from three species.

The disruption of sex determination in gonochoristic animals after genome duplication is considered the main reason for the relatively rare occurrence of polyploid animals compared to plants [52,53]. In vertebrates, in which autotetraploidy may be rarer than (hybrid-origin) allopolyploidy [54], polyploidy occurs especially frequently in amphibians [55], only few of which have their mostly undifferentiated sex chromosome characterized. In clawed frogs (*Xenopus*), alloploidy ranges from diploid to do-decaploid (12n) and subgenome evolution in allopolyploids has only recently been studied [56,57]. In *Xenopus laevis*, the female-determining gene *DM-W* is a paralog of *DMRT1* [58,59], and arose after (and perhaps in response to) tetraploidization [60–62]. However, it is also found in some related *Xenopus* [60,61] but not in the entire radiation [63,64]. The clade-wide conservation of a male heterogametic sex determination gene and system (but not chromosome) in the 50 Myr-old, ancient tetraploid family Salmonidae (see above) curiously also evolved in a system with a common ancestral polyploidization event, as suggested here for Acipenseridae, with apparently conserved female heterogamety.

Our finding is too recent to allow comprehensive interpretation. Whole-genome approaches are underway to address several of the most pressing questions, such as conservation of the region or the entire sex chromosomes in sturgeons. Conservation of the latter would even more strongly challenge the classical paradigm of sex chromosome evolution [65–67] than the 180 Myr-long conservation of this small sex-linked sequence.

### (a) Reasons for the challenges to identify a female-specific genomic region in sturgeon

The search for a sex chromosome or sex-linked markers in sturgeons has been unsuccessful for a long time [36,48,49]. We discuss four major challenges, why this has been the case.

First, in *A. ruthenus*, the identified W-specific sequence is very short and resides on a large undifferentiated chromosome. It comprises only approximately 16 kb, corresponding to 0.001% of the genome or 0.02% of the corresponding chromosome (approx. 106 Mb). It is impossible to detect such a small structural difference by karyotype imaging methods unless the W sequence is known and FISH probes can be designed.

Second, the high repeat content of the W-specific locus (approx. 43%; www.repeatmasker.org) complicates its assembly by short-read data alone. Thus, when reconstructing female-specific genomic regions from unmapped pool-seq data, we filtered female-specific reads and independently assembled them. Unfortunately, the resulting small contigs were of limited utility for female-specific primer design. Looking for an explanation, we mapped these contigs back to the female genome assembly and found that only 0.09% of the sequence (a single contig) matched the W region, which we have identified by a sequencing coverage analysis.

Third, short-read-based methods that call and analyse variants between female and male sequencing data suffered from the low number of sex-specific variants in sturgeons (a few hundreds to thousands depending on filter parameters), especially when reduced representation methods like RADseq are used [32]. Even our whole-genome pool-sequencing approach was unable to define the W-specific region by SNP markers. This approach only uncovered a nearby region (distance 1.16 Mb, approx. 1% of chromosome length); a result that could point to reduced recombination around the W-specific sequence (W-linked SNPs). Finally, analysis of sequencing coverage enabled identification of the W-specific locus in *A. ruthenus*, but detection of haploid/diploid coverage differences was still complicated due to high variability (electronic supplementary material, figure S2), causing noisy signals for haploid genomic regions. Completely deleted regions in one sex compared to the other provided significant signals, but rely on the availability of a high-quality genome assembly of the heterogametic sex (figure 1).

### (b) Potential application of the W-specific marker for conservation measures, aquaculture production and ethical issues

Beyond the basic biological interest in this ancient sex determination mechanism, molecular sexing of sturgeons is of great relevance in studies on wild sturgeon populations, their conservation and conservation aquaculture as well as caviar-producing aquaculture. Currently, ultrasonic diagnosis or biopsies are used for sexing. For ultrasonic sexing to become reliable, sturgeons have to reach progressed stages of maturation—6–10 years in some species—while biopsies are invasive and stress or even harm the fish [68]. Our molecular marker's reliability outperforms methods like early biopsies or ultrasound-sexing. Future application of cotton skin swabs will make molecular sexing much less stressful for the fish. It will also aid conservation by reducing time and efforts to rear fish intended as future broodstock in *ex situ*-programmes, reducing costs and improving the selection of candidates for living gene banks, not only based on rare alleles but also on sex. In this case, fish not selected for broodstock development can be released in the frame of recovery programmes. In addition, in the caviar aquaculture, females, which are commercially more attractive, can be selected early on, and differentiated rearing for caviar (females) and meat (males) production becomes feasible. Releases of commercially reared fish are no option in conservation programmes since they show restricted fitness due to intensive aquaculture [69]. In any case, we strongly emphasize that the early selection of females must not lead to littering male sturgeons, since their meat is a valuable source of protein [70].

## Data accessibility

The pool-sequencing Illumina reads are available in the SRA (SRX9341540 and SRX9341541), under BioProject reference PRJNA670610; all other datasets supporting this article have been uploaded as part of the electronic supplementary material.
